# Classifying Driving Fatigue by Using EEG Signals

**DOI:** 10.1155/2022/1885677

**Published:** 2022-03-24

**Authors:** Changqing Zeng, Zhendong Mu, Qingjun Wang

**Affiliations:** ^1^School of Software, Nanchang University, Nanchang 330047, Jiangxi, China; ^2^The Center of Collaboration and Innovation, Jiangxi University of Technology, Nanchang 330098, Jiangxi, China; ^3^College of Economics and Management, Shenyang Aerospace University, Shenyang 110136, Liaoning, China; ^4^Nanjing University of Aeronautics and Astronautics, Nanjing 210016, China

## Abstract

Fatigue driving is one of the main reasons for the occurrence of traffic accidents. Brain-computer interface, as a human-computer interaction method based on EEG signals, can communicate with the outside world and move freely through brain signals without relying on the peripheral neuromuscular system. In this paper, a simulation driving platform composed of driving simulation equipment and driving simulation software is used to simulate the real driving process. The EEG signals of the subjects are collected through simulated driving, and the EEG of five subjects is selected as the training sample, and the remaining one is the subject. As a test sample, perform feature extraction and classification experiments, select any set of normal signals and fatigue signals recorded in the driving fatigue experiment for data analysis, and then study the classification of driver fatigue levels. Experiments have proved that the PSO-H-ELM algorithm has only about 4% advantage compared with the average accuracy of the KNN algorithm and the SVM algorithm. The gap is not as big as expected, but as a new algorithm, it is applied to the detection of fatigue EEG. The two traditional algorithms are indeed more suitable. It shows that the driver fatigue level can be judged by detecting EEG, which will provide a basis for the development of on-board, real-time driving fatigue alarm devices. It will lay the foundation for traffic management departments to intervene in driving fatigue reasonably and provide a reliable basis for minimizing traffic accidents.

## 1. Introduction

The fast-paced life of modern society not only promotes the rapid development of society, but also increases the work pressure of workers. Long-term high-intensity work can easily cause workers' mental fatigue. Mental fatigue will not only reduce work efficiency, but also be harmful to health and even cause accidents. In recent years, fatigue driving has become one of the main causes of traffic accidents. Truck drivers who travel long distances and drivers who work overtime are often prone to fatigue or drowsiness and some even fall asleep while driving. Therefore, fatigue detection has become an important topic in the field of driving safety. Scientists try to use this, to try to reduce the frequency and severity of traffic accidents, and formulate relevant regulations.

At present, there are still many problems in the research on driving fatigue detection technology. For example, the method of detecting the physical reaction of the driver has a high false alarm rate; the method of detecting vehicle parameters requires clear markings on the center line of the road and good light; and the method of measuring the pupil diameter is difficult to implement. In recent years, a large number of scientific researchers have invested in the research of noncontact collection of ECG and EEG and have made great breakthroughs, making the detection of fatigue through biological signals extremely attractive. It provides a basis for further research and development of on-board, real-time driving fatigue alarm devices, and provides a reliable basis for traffic management departments to scientifically and reasonably intervene in driving fatigue and minimize man-made traffic accidents.

At present, the mental fatigue detection at home and abroad mainly selects the following three characteristics:

1. Behavioral characteristics: behavioral characteristics include changes in lateral lane positions and variability in vehicle heading differences, which are one of the commonly used driving indicators to identify fatigue through vehicle behavior. This method is easy to understand, but the standards for these features have not yet been formed, and it is not easy to achieve stable and effective recognition results.
2. Facial expression characteristics: facial expression characteristics include the degree of eye closure and the frequency of nodding, etc.
3. Although this method is very convenient to use, the detection of these features is affected by various factors, such as image angle and image brightness. These limitations reduce the overall recognition accuracy of this method and limit its application scenarios.
4. Physiological characteristics: physiological characteristics include ECG, heart rate, electromyogram, and EEG. Different from behavioral features and facial features, these features, as the mapping of the physiological changes of the human body, objectively reflect the current state and environment of the human body. Therefore, driving fatigue detection based on physiological features is a very popular application direction. And among these fatigue detection methods based on physiological features, detecting EEG signals is considered the most direct, effective, and promising method to detect driving fatigue.

Fatigue refers to a complex physiological phenomenon in which the physiological and psychological functions of the human body are out of balance due to continuous or high-intensity physical or mental work. Driving fatigue is the driver's excessive consumption of continuous driving or other physical labor for a long time, or lack of sleep, which leads to drowsiness, sluggishness, and weakness in the limbs. phenomenon of the situation. When a driver is fatigued, many features will change, including visible features such as driving posture, blink rate, eye closure time, nodding action, reaction speed, facial expressions, and invisible features such as EEG, ECG signal, EMG signal, skin temperature, skin resistance, respiratory rate, etc. The main manifestations are drowsiness and weakness of the driver, difficulty in eliminating drowsiness, decreased vision, gradually narrowing of the field of vision, inattention, decreased judgment, decreased thinking ability, weak limbs, decreased sensory organ function, unstable driving movements, delayed judgment, Rhythm disorders, knee fatigue, neck stiffness, backache and leg pain, loss of self-control, resulting in a series of uncomfortable symptoms such as being easily excited, irritable or driving fast.

When the driver is slightly fatigued, the shifting is not timely and accurate. In moderate fatigue, the operation is sluggish and sometimes even forgets to operate, In severe fatigue, often subconscious operation or short-term sleep phenomenon, loss of control over the vehicle.

In recent years, how to recognize the fatigue state of a driver has become a hot topic today. Chain *R* defines an EEG-based driver fatigue test method. The results show that the frequency characteristics of EEG can be well applied to driving fatigue testing, but the overall study lacks data support, and more data is needed to support its conclusions [1]. Yimyam W used 50 recorded EEGs to participate in driving simulation experiments, resulting in two fatigue states: alertness and drowsiness. The experimental results show that as the fatigue degree increases, the two complexity parameters are significantly reduced. The results show that these two nonlinear indicators can be used to characterize driver fatigue. However, the lack of experimental data in his research led to small differences in sample sets, resulting in inaccurate results [2]. Zeng studied the complex, unstable, and nonlinear characteristics of evaluating EEG signals. The results show that: this paper uses the combination of finite element features and AdaBoost classifier to detect driver fatigue based on EEG, ensuring the confidence to explore the internal physiological mechanism and wearable applications. The experiment did not take into account the deviation of the subject's gender and age parameters in the experiment [3].

This paper first extracts more representative EEG signal features by designing a more appropriate feature extraction method, and then selects a suitable classifier to perform optimized to achieve a better classification effect, and classify the signal features extracted by the new feature extraction method through the optimized classifier to achieve the optimal classification effect of the experiment. When studying driving fatigue, this article continuously analyzes the EEG data of driving for 2 hours, trying to find the time when the fatigue occurs. This is something that scholars who have studied driving fatigue have never done before. Most of them only select data from fatigue and normal states to analyze the differences.

## 2. EEG Signals and the Classification of Driver Fatigue

### 2.1. Composition and Classification of Brain-Computer Interface

The exploration of the brain's operating mechanism is not only of great significance in medicine, but also of great help to the development of artificial intelligence. Brain-computer interface is a new technology developed for this purpose, and its application in scientific research is becoming increasingly extensive.

#### 2.1.1. System Composition of Brain-Computer Interface

The core of brain-computer interface (BCI) technology is to directly collect the EEG of the cerebral cortex through the electrodes and transmit it to the computer via the circuit for signal analysis, and then send the corresponding control commands to the external equipment to achieve a complete EEG control path, instead of passing through normal neuromuscular pathways such as peripheral nerves and muscle tissues [4, 5]. The main working principle of the brain-computer interface system is: nerve cells generate local potentials when they are active. These potentials can be collected and analyzed in the cortex to analyze the changes in the brain's thinking activity at this time. These changes can be recorded and amplified by the electrode cap. After processing, the collected EEG extraction features are analyzed through information analysis, and the corresponding control instructions are made according to the features to complete the control of the external equipment. The pattern recognition part of BCI is mainly to send the EEG characteristic signal to the classifier and to distinguish the classification space to which the characteristic signal belongs by searching for a suitable decision plane. The result of the classifier is directly related to whether the feature is correctly identified, and therefore directly affects the effect and performance of the BCI. Different classifiers have different advantages in different application scenarios because of their different algorithm principles, so the choice of classifier is essential [6, 7].

A general brain-computer interface system consists of the following five main steps: EEG signal acquisition, signal preprocessing, feature extraction, pattern recognition, and sending external control commands.

The signal acquisition part of BCI is the connection between the system and the brain. This part mainly collects the electrical signals of the cerebral cortex through the electrode cap, then amplifies the electrical signals, and converts the amplified EEG signals into digital signals through A/D conversion. The signal acquisition part of BCI mainly involves the selection of signal types and the number of channels and positions. Since the collected EEG signals will be interfered by EEG, ECG, power, frequency interference, and some high-frequency noise signals, these signals will affect the follow-up, the reliability and accuracy of the processing part. Therefore, the preprocessing part of BCI will use denoising algorithm or filter to remove the noise signal doped in the signal and improve the signal-to-noise ratio of the EEG signal.

EEG signals are used by most BCI systems due to their high temporal resolution. BCI has a wide range of applications in the field of artificial intelligence because of its cheap equipment and no harm to the human body. The BCI system has a variety of classification methods from different perspectives:

Lossy and lossless BCI systems, the acquisition methods of the lossy BCI system can be divided into implantable and semi-implantable. The feature of the implantable BCI system is that the electrodes are implanted into the brain tissue in the cranial cavity, so that the collected brain signals are more accurate; the feature of the semi-implantable BCI system is that the electrodes are attached to the cerebral cortex in the cranial cavity, and the brain Cortical potential changes can be accurately recorded by the electrodes.

Induced and spontaneous BCI systems, the evoked BCI system has many kinds of elicitation methods, which can be divided into exogenous stimulation and endogenous stimulation according to the different stimulation sources. Potential changes induced by exogenous stimuli are called exogenous evoked potentials, including visual stimulus evoked potentials, auditory stimulus evoked potentials, and tactile stimulus evoked potentials. Endogenous stimuli are related to cognitive processes in the brain, and endogenous evoked potentials are brain potentials evoked by specific, multiple or diverse stimuli.

#### 2.1.2. Application Fields of Brain-Computer System

*(1) Rehabilitation Field*. The BCI system has the most urgent needs in the field of rehabilitation. There are many patients with physical disabilities throughout the country. For them, the BCI system provides them with a new channel for communication with the outside world, which can significantly improve their quality of life and even live a normal life.

*(2) Military Field*. Although the current research on related technologies has not reached the stage of maturity in movies, the concept of using thinking activities in military operations is the consensus of all countries in the world, many countries have spent a lot in this field, and movies have also been realized.

*(3) Leisure and Entertainment Field*. Leisure and entertainment have always been an indispensable part of human life, so the research direction of brain-computer interface is also biased towards this [8]. Now, ordinary keyboard and mouse devices can no longer keep up with people's response speed and computer processing speed. People urgently need a new control method to control computers more conveniently for entertainment activities. Simple such as browsing the web, typing on the Internet, designing drawings, complex multiline operations, controlling games, These applications will greatly change the way of life and entertainment in the future, have large market potential, and attract countless teams to develop related technologies.

*(4) The Field of Brain Science Research*. The research of the BCI system originally originated from the study of brain cognitive science, such as the use of brain electricity to detect polygraphs, the study of mental activity based on brain electricity, and this article involved in driving fatigue detection based on BCI system [9]. The research of the BCI system is based on the research of brain science, and the research of the BCI system in turn provides help to the research of brain cognitive science.

### 2.2. EEG Signal

Generally speaking, EEG refers to the electrophysiological phenomenon produced by the activity of brain nerve cells in the cerebral cortex, which is a kind of bioelectric signal. Since the human body is a complex system, the electrical signals collected through the scalp include not only EEG, but also physiological signals such as my electricity and ocular electrical signals, as well as noise interference such as instrument power frequency interference, so the effect of directly processing the collected EEG is not ideal. It is necessary to preprocess the EEG and then conduct research and analysis [10, 11].

People will experience various subjective feelings when driving fatigue, such as dizziness, blurred vision, difficulty concentrating, feeling irritable, feeling sleepy, muscle weakness, reference basis. When driving fatigue, the subjects will have many weakened functions controlled by the brain. Different parameters of the EEG signal represent different activities of the human brain and different mental states. However, the accurate extraction of these parameters requires good technical means suitable for EEG signals.

#### 2.2.1. The Structure and Function of the Brain

**Figure 1:**
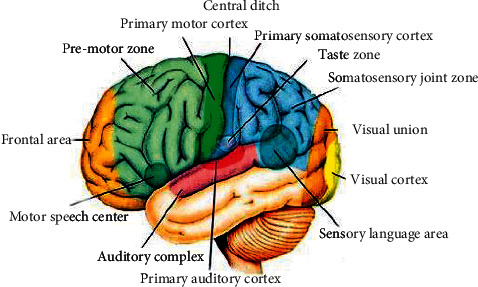
Distribution map of cerebral cortex function.

The brain is the most complex and mysterious organ in the human body, and there are many mysteries that have not yet been completely solved. The brain is divided into two parts: the left brain and the right brain. According to the existing medical data, the left and right hemispheres are responsible for different functions such as thinking and imagination, and are connected to each other through the corpus callosum. The left and right hemispheres are subdivided into four parts: frontal lobe, parietal lobe, temporal lobe, and occipital lobe. These areas control different functions. According to some latest brain science research data, the various functions of the brain are not only completed by the abovementioned parts alone, but are the result of the collaborative processing of various parts of the brain [12–14]. Even after some parts are damaged, the brain can coordinate the other parts to complete the functions of the damaged part of the brain. The cerebral cortex is the general term for the gray matter of the brain's surface layer, and its thickness is about 1.5–5.0 mm. The gray matter of the cerebral cortex in different regions corresponds to different functions, as shown in Figure 1.

According to scientific research data, at this stage, the cerebral cortex contains tens of billions of nerve cells. With this large group of nerve cells, the brain can achieve very complex functions. For driving fatigue detection based on EEG, it is first necessary to understand the relationship between mental fatigue and the state of different areas of the cerebral cortex and time, to accurately judge the state of mental fatigue.

#### 2.2.2. The Generation Mechanism of EEG Signals

Generally speaking, EEG refers to the local electrical signal produced by the activity of nerve cells in the cerebral cortex, which is produced by the electrophysiological activity of a large number of nerve cells in the relevant areas of the brain. According to the research data, the dendrites of nerve cells will generate an action potential after receiving stimulation. The potential will be transmitted along the axon to the axon terminal, and the potential will be transmitted to the next nerve cell through the axon terminal. The electrical signal generated by these large nerve cells transmitting potential is EEG [15, 16].

#### 2.2.3. Characteristics and classification of EEG signals

1. Randomness: because the cerebral cortex is not completely insulated, the electrical signals of different areas will influence and interfere with each other, sometimes accompanied by unconscious light discharge behaviors. Therefore, EEG has significant randomness, which humans cannot completely present. Analyzing the trend of EEG can only roughly analyze the characteristics of EEG.
2. Nonstationary: the generation of brain signals is affected by many factors, so it has the characteristics of nonstationary. Using this feature of EEG, the amplitude and frequency of EEG can be compared to distinguish whether the brain is in a state of fatigue.
3. What we usually call an EEG refers to the brain scalp EEG, which is actually a graph of the relationship between the scalp potential difference and time. Brain waves are the comprehensive external manifestations of the overall activity of brain nerve cells, including ion exchange and metabolism. The EEG signal is an electrical phenomenon displayed on the surface of the cerebral cortex or scalp by the physiological activity of brain nerve cells. This electrical phenomenon accompanies a person's life from the beginning to end. Once a person dies, the electrical phenomenon disappears. Therefore, the EEG signal is a signal obtained by extracting the spontaneous and rhythmic physiological activity of the brain cell population on the surface of the scalp and amplifying it by a million times. EEG signals can be divided into spontaneous EEG and evoked EEG. Spontaneous EEG refers to changes in EEG without external stimulation. Induced EEG is a change in brain potential caused by artificially stimulating human sensory organs.
4. Nonlinearity: the generation of EEG is based on the multiple coupling of human physiological state and life phenomena, so it has chaotic characteristics and nonlinearity. Simply using linear analysis of EEG cannot fully reflect the characteristics of EEG. It is necessary to use nonlinear analysis EEG to better analyze the internal information of EEG [17].
5. Low amplitude, low frequency, and low signal-to-noise ratio: EEG signals have the characteristics of low amplitude, low frequency, and low signal-to-noise ratio. Since the EEG signal is the bioelectricity generated by the activity of nerve cells, the amplitude of the EEG signal is relatively low only at the micro- and millivolt level, generally not exceeding 100 *μ*V. The frequency of EEG signals is relatively low, generally between 0.5 and 100 Hz. It is precisely because of the characteristics of low amplitude and low frequency of EEG signals that it is easily interfered by other external signals and has the characteristics of low signal-to-noise ratio.

EEG signal is a manifestation of brain nerve activity and an important signal reflecting the state of brain activity. Its amplitude range is one and its frequency range is one. The rhythm of EEG signals changes with the change of a person's mental state and a series of transients such as sharp waves or spikes in epilepsy patients also appear. Usually, the collected EEG signals are very weak, and there is a lot of background noise and strong interference signals, such as power, frequency interference, ECG interference, breathing interference, swallowing, eye blinking, which increase the difficulty of analysis. However, because the method is a noninvasive method and contains a lot of physiological and pathological information, it still attracts the interest of many researchers. They focus on the time-varying nonstationary characteristics of EEG signals. It has continuously proposed analytical methods that can effectively characterize different physiological states of the brain [18, 19].

### 2.3. Feature Extraction Based on Frequency Band Power Spectrum

When the human brain is active, it produces five frequency bands: alpha, gamma, theta, delta, and sigma. When the power spectrum peaks of the alpha wave and the beta wave are obvious, which are the main bands of the EEG, it indicates that the subject is awake; when the subject feels tired or even sleeps the peaks of theta wave and delta rise significantly [20, 21]. Using the STFT algorithm to extract the power spectrum is a power spectral density function (PSD) feature as follows:

(1) $$\begin{array}{c} STFT\left\{x\left[n\right]\right\}\left(m,w_{k}\right)=\sum_{n=1}^{N}x\left[n\right]w\left[n-m\right]e^{-jw_{k}n}, \end{array}$$

where *w*_*k*_=(2*πk*/*N*),  *k*=0,1,…, *N* − 1 is the angular frequency, *m* is discrete and *?* is continuous, its mathematical expression is as follows:

(2) $$\begin{array}{c} w\left[n\right]=0.5\left[1-\cos\left(\frac{2\pi n}{\left(N-1\right)}\right)\right]=\;\sin^{2}\left(\frac{\pi n}{\left(N-1\right)}\right). \end{array}$$

The energy spectrum at different frequencies of different EEG frequency bands is calculated as follows:

(3) $$\begin{array}{c} E\left(w_{k}\right)=X\left(m,w_{k}\right)X^{*}\left(m,w_{k}\right),\\ PSD\left(w_{k}\right)=\frac{1}{N}E\left(w_{k}\right),\;k=0,1,\ldots ,N-1. \end{array}$$

The energy spectrum of each EEG band can then be defined as:

(4) $$\begin{array}{c} E\left(w\right)=\sum_{k=1}^{n}E\left(w_{k}\right), \end{array}$$

where *x*[*n*]={*x*_1_, *x*_2_,…, *x*_*n*_} represents all frequencies in a frequency band.

### 2.4. Feature Extraction Method Based on EMD and Energy Spectrum

#### 2.4.1. Empirical Mode Decomposition

Empirical Mode Decomposition (EMD) decomposes a given signal *x*(*t*) into a series of Intrinsic Mode Function (IMF) components from high to low. EMD can be regarded as a type of wavelet decomposition. Its subbands are established according to the decomposition of each component of the signal. Each IMF component generated represents the details of the signal on a certain scale and frequency band [22, 23]. The steps of EMD decomposition are as follows:

(1) Identify all maximum points in the EEG and fit the upper envelope *e*_up_(*t*) of the signal, and identify all minimum points in the EEG and fit the lower envelope *e*_low_(*t*) of the signal.
(2) Calculate the average value *m*_1_(*t*) according to the synthesized upper and lower envelopes, the formula is
  (5) $$\begin{array}{c} m_{1}\left(t\right)=\frac{e_{\text{up}}\left(t\right)+e_{\text{low}}\left(t\right)}{2}. \end{array}$$
(3) Subtract *x*(*t*) from *m*_1_(*t*) to obtain *h*_1_(*t*), and use the obtained *h*_1_(*t*) as the new EEG *x*(*t*). Repeat step 1). After *k* screening, *h*_1_(*t*) at this time is the first IMF component of the signal, denoted as *c*_1_(*t*)=*h*_1_(*t*).
(4) Repeat the above steps with the difference *r*_1_(*t*)=*x*(*t*) − *c*_1_(*t*) between *c*_1_(*t*) and *x*(*t*) as a new signal to obtain a series of IMF components. When the obtained IMF component or residual signal is less than a preset value, the empirical mode decomposition step is completed.

#### 2.4.2. Principle of Energy Spectrum

Energy spectrum (Energy Spectral Density, ESD), arrangement of the harmonic energy contained in the signal, is called the energy spectrum, which describes the energy contained in each frequency component in the signal. The formula of the energy spectrum is as follows:

(6) $$\begin{array}{c} \Phi \left(w\right)=\left|\frac{1}{\sqrt{2\pi }}\sum_{n=-\infty }^{\infty }f_{n}e^{-jw_{k}n}\right|\\ =\frac{F\left(w\right)F^{*}\left(w\right)}{2\pi }. \end{array}$$

In the formula, *f*_*n*_ is the signal sequence. Divide the observation data x(t) of length N into K segments of data of length *M*, that is, *N*=KM. Estimate the third moment of each piece of data *x*_i_(t) (*i* = 1,2, ..., k):

(7) $$\begin{array}{c} \hat{r}\left(i\right)\left(m,n\right)=\frac{1}{M}\sum_{k=s_{1}}^{s_{2}}x_{i}\left(t\right)x_{i}\left(t+m\right)x_{i}\left(t+n\right),\;i=1,2,\ldots ,k. \end{array}$$

Among them *S*_1_=max(1,1 − *m*, 1 − *n*), *S*_2_=min(*M*, *M* − *m*, *M* − *n*). Find the overall average of the third-order moment estimates of these K segments of the data, and find the third-order cumulant estimate of x(t):

(8) $$\begin{array}{c} \hat{r}\left(m,n\right)=\frac{1}{K}\sum_{i=1}^{k}{\hat{r}}^{\left(i\right)}\left(m,n\right). \end{array}$$

By estimating the third moment $\hat{\beta }$ of the non-Gaussian white noise sequence w(k), the parameters of the AR model are obtained:

(9) $$\begin{array}{c} \hat{r}\cdot a=\hat{b},\\ \hat{r}=\left[\begin{array}{cccc} \hat{r}\left(0,0\right) & \hat{r}\left(1,1\right) & \cdots & \hat{r}\left(p,p\right)\\ \hat{r}\left(-1,-1\right) & \hat{r}\left(0,0\right) & \cdots & \hat{r}\left(p-1,p-1\right)\\ \vdots & \vdots & \hat{r}\left(0,0\right) & \vdots \\ \hat{r}\left(-p,-p\right) & \hat{r}\left(-p+1,-p+1\right) & \cdots & \hat{r}\left(0,0\right) \end{array}\right],\\ \hat{b}={\left[\hat{\beta },0,0,\ldots ,0\right]}^{T}. \end{array}$$

A is the AR model parameter to be estimated for *p*. Use *p* model parameters to estimate the energy spectrum:

(10) $$\begin{array}{c} B\left(w_{1},w_{2}\right)=\hat{\beta }\hat{I}\left(w_{1}\right)\hat{I}\left(w_{2}\right){\hat{I}}^{*}\left(w_{1}+w_{2}\right). \end{array}$$

Among them,

(11) $$\begin{array}{c} \hat{I}\left(w\right)={\left[1+\sum_{n=1}^{p}a_{n}\exp\left(-jwn\right)\right]}^{-1},\;\left|w\right|\leq \pi . \end{array}$$

#### 2.4.3. Feature Extraction Based on EMD and Energy Spectrum

**Figure 2:**
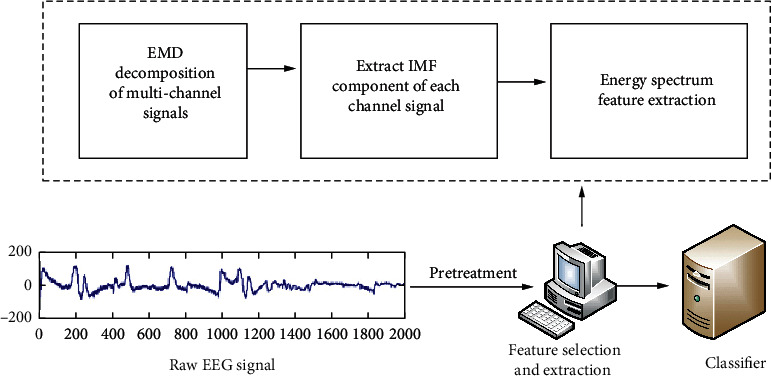
Flow chart of feature extraction algorithm.

Traditional EMD-based feature extraction methods usually contain a large number of time-domain signals, which is very helpful for extracting the main components of the signal, but it is not ideal for the extraction of fatigue signals. The energy spectral density function can extract the frequency domain information of each IMF component and extract the time-frequency information of EEG by combining it with EMD decomposition. The flow chart of the algorithm is shown in Figure 2.

It can be seen from Figure 2 that after the original EEG has been preprocessed, the irrelevant noise signals have been roughly filtered out. The first three layers of IMF components are selected for processing, and after feature extraction, 96 energy spectrum features (32 channels × 3 layers of IMF) are obtained. Compared with the power spectrum feature extraction method, it has fewer features and requires less calculation and calculation time for classification.

## 3. Experimental Design of Driver Fatigue Classification Based on EEG Signal

### 3.1. Simulated Driving Experiment Design

The EEG signals in this experiment are all collected by laboratory simulation driving. In this article, the real driving process is simulated by using a driving simulation platform composed of driving simulation equipment and driving simulation software. The driving simulator platform is an advanced simulator system that can simulate real driving conditions while providing dynamic displays of vehicles and surrounding traffic. The driving simulation platform itself consists of a fixed car, steering wheel, brake pedal, and accelerator pedal, a large screen, a high-performance computer, driving simulation software, and a multifunctional data acquisition board. The platform can dynamically record EEG during the mission and calculate the driving and running status of the vehicle.

**Figure 3:**
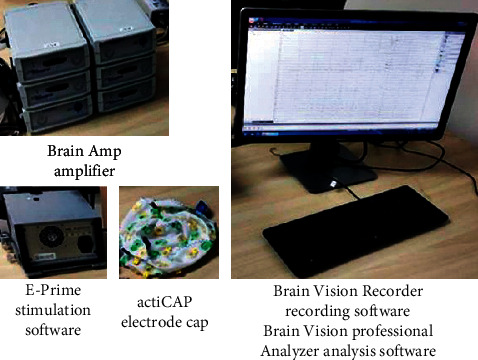
EEG acquisition system(https://image.cnki.net/, author authorization is available).

The EEG acquisition equipment used in this experiment is an EEG signal acquisition and analysis system composed of Brain Amp, DC amplifier of German Brain Products, and corresponding software. The system includes E-Prime stimulation software, actiCAP electrode cap, Brain Amp amplifier, Brain Vision Recorder recording Software, and Brain Vision professional Analyzer analysis software, the physical object of the equipment is shown in Figure 3.

### 3.2. Experimental Objects and Methods

In this experiment, the EEG data of 5 participants was selected as the training set, and the remaining 1 data was used as the test sample for feature extraction and classification experiments, pick out two sets of data from the data, one is normal signal data, and the other is fatigue signal data, and analyze them. We asked the participants to sleep only 4 hours as fatigue state data the night before data collection, and 8 hours sleep as waking state data, to obtain EEG data in the two states.The participants' simulated driving time was set at 8 pm the next day, during which EEG recording was started at 8 : 10, and signal recording was stopped at 8 : 30 pm.

**Figure 4:**
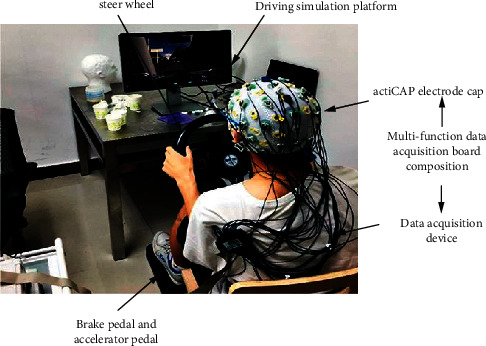
Simulated driving experiment diagram. (https://image.cnki.net/, author authorization is available).

The EEG acquisition experiment took 20 minutes. During the experiment, the subject was asked to sit on a chair and simulate the driving process on the driving simulation platform. During the experiment, the EEG was recorded and used with a 32-channel electrode cap according to the international 10–20 lead system. Brain Vision Recorder records EEG with a sampling rate of 1000 Hz. The temperature of the laboratory is maintained at 22°C, which provides the subjects with a comfortable and stable experimental environment.  Figure 4 shows the actual scenario of the experiment.

### 3.3. EEG Signal Collection

Due to the different functional composition of various regions of the brain, the EEG signal bands appearing in different regions will also change. In addition, EEG signals have the characteristics of low amplitude and low frequency, which are easily disturbed by noise signals. Therefore, how to choose appropriate electrode selection location is essential. Using Brain Vision Recorder recording software to record the EEG during driving, it can be seen that most of the electrodes are in good contact with the scalp, and all signals meet the requirements of EEG collection. The EEG of fatigue and normal driving were collected, respectively. Each subject collected 20 minutes of normal signal and 20 minutes of fatigue signals. The signals collected every 10 seconds were used as a sample to construct training samples and test samples. In this experiment, six subjects collected a total of 720 groups of normal EEG samples and 720 groups of fatigue EEG samples, and the collected 1440 groups of EEG data samples were subjected to subsequent analysis and processing.

### 3.4. Frequency Reduction and Filtering of EEG Signals

To study the driving fatigue EEG, it is necessary to analyze the five main EEG bands of alpha, beta, gamma, theta, and delta. The frequency bands of these five signals are roughly between 0.1 and 50 Hz, which belong to low-frequency signals, and the acquisition equipment, sampling frequency is 1000 Hz, which not only carries a large amount of irrelevant high-frequency noise and interferes with data processing, but also a large amount of data processing also causes a lot of burden on the processing and operation of the brain-computer interface device. Therefore, to better carry out the subsequent processing, the collected EEG is first subjected to frequency reduction processing, and the sampling frequency is reduced to 200 Hz, which greatly reduces the amount of data calculation and speeds up the data processing.

### 3.5. Denoising of EEG Signals

#### 3.5.1. Hard Threshold and Soft Threshold Denoising

Noise shows strong randomness in the wavelet domain and can be considered as Gaussian noise. According to the characteristics of Gaussian distribution, most of the noise coefficients are in the interval [-3*σ*, 3*σ*]. Therefore, as long as the threshold is set to *λ* = 3*σ*, most of the noise can be removed. Among them, the hard threshold wavelet denoising formula is as follows:

(12) $$\begin{array}{c} {\tilde{d}}_{j,k}=\left\{\begin{array}{c} d_{j,k},\left|d_{j,k}\right|\geq \lambda ,\\ 0,\left|d_{j,k}\right|<\lambda . \end{array}\right) \end{array}$$

Among them, *d*_*j*,*k*_ is the wavelet coefficient, ${\tilde{d}}_{j,k}$ is the estimated value of the wavelet coefficient after hard threshold denoising, and *λ* is the threshold.

However, directly removing these wavelet coefficients will cause abrupt changes in the wavelet domain, resulting in local jitter in the denoised signal, which will affect the analysis and processing of the signal. Therefore, a soft threshold wavelet denoising method is proposed. Subtract 3*σ* from the coefficients greater than 3*σ*, and add 3*σ* to the coefficients less than -3*σ*. The formula of the soft threshold wavelet transform denoising method is as follows:

(13) $$\begin{array}{c} {\tilde{d}}_{j,k}=\left\{\begin{array}{c} d_{j,k}-\lambda ,d_{j,k}\geq \lambda ,\\ d_{j,k}+\lambda ,d_{j,k}\leq -\lambda ,\\ 0,\left|d_{j,k}\right|<\lambda . \end{array}\right) \end{array}$$

After soft threshold processing, the signal after denoising is smoother than that after hard threshold denoising. At the same time, it will also cause partial loss of detail signal.

#### 3.5.2. Improve Soft Threshold Denoising

The improved soft threshold method combines the advantages of soft and hard threshold methods and can effectively remove low-frequency noise. The formula is as follows:

(14) $$\begin{array}{c} {\tilde{d}}_{j,k}=\left\{\begin{array}{c} a\lambda^{2}-\lambda +d_{j,k},d_{j,k}\geq \lambda ,\\ ad_{j,k}^{2},0<d_{j,k}<\lambda ,\\ -a\lambda^{2}+\lambda +d_{j,k},d_{j,k}\leq -\lambda ,\\ -ad_{j,k}^{2},-\lambda <d_{j,k}<0, \end{array}\right),\;a<\frac{1}{\lambda }. \end{array}$$

Among them, a is the shape coefficient. The value of a can control the shape of the function in the *d*_*j*,*k*_ < *λ* area, that is, the degree of attenuation. When the value is 0, the formula for improving the soft threshold method is equivalent to the soft threshold method. The selection formula of the threshold is

(15) $$\begin{array}{c} \lambda_{j}=\frac{\sigma \sqrt{21g\left(N\right)}}{\mathrm{lg}\left(j+1\right)}, \end{array}$$

where *b* ≥ 0, *j* is the number of decomposed layers, N is the number of wavelet transform coefficients corresponding to the *j* layer, *λ*_*j*_ is the wavelet threshold, *s* is the variance of the noise, and in real life, the way the noise is generated is various, so the value of *s* is generally estimated according to the first-level wavelet packet decomposition coefficient, the estimation formula is as follows:

(16) $$\begin{array}{c} \hat{\sigma }=\frac{\sum_{k=1}^{N}\left|d_{1k}\right|}{0.6745*N}. \end{array}$$

Among them, d_1k_ is the wavelet decomposition coefficient of the first wavelet decomposition layer, and 0.6745 is the adjustment coefficient of Gaussian white noise.

## 4. Experimental Driver Fatigue Classification Based on EEG Signal

### 4.1. Compare the Denoising Effects of Different Algorithms

**Figure 5:**
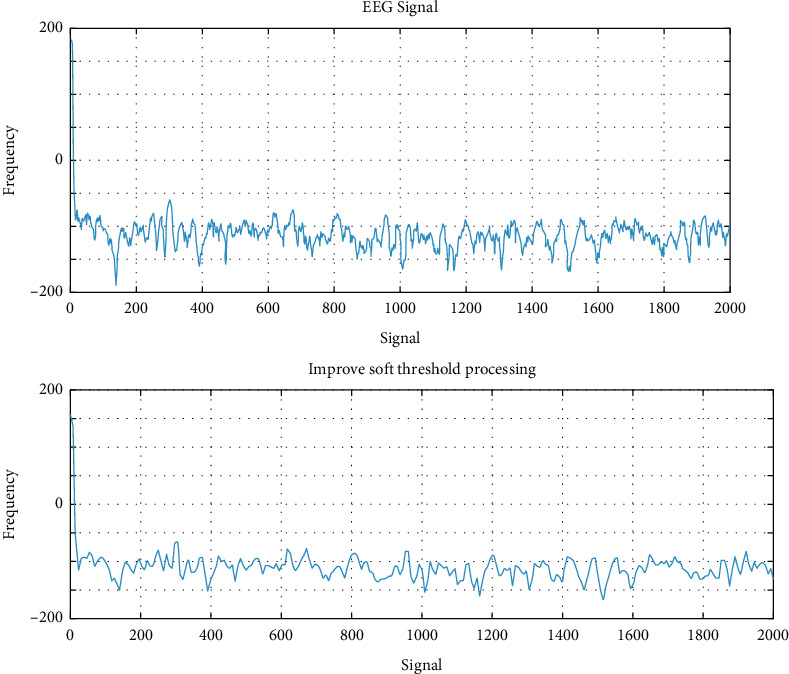
Experimental comparison of three wavelet threshold denoising methods.

We tested the effect of the improved method, the signal used is a piece of data from the Fp1 channel of subject No. 1. The sampling frequency after frequency reduction is 200 Hz, and it has passed the bandpass filter of [0.1 Hz, 50 Hz]. Processing. The wavelets in the experiment all decompose the original signal in three layers and extract coefficients. The shape coefficient of the soft threshold is 0.01 and the scaling factor is 0.5. The result is shown in Figure 5.

It can be seen from Figure 5 that there are many glitches in the signals of the hard threshold denoising and soft threshold denoising methods, therefore, the signal of the improved method is clearly better than the other three signals. Although the hard threshold denoising method and the soft threshold denoising method have certain effects, the signal effect after the improved soft threshold denoising processing is the best. This shows that the improved soft threshold denoising method can effectively combine the traditional hard threshold and soft threshold. Advantages of the method.

Want to quantitatively analyze the pros and cons of the three wavelet threshold denoising methods, the signal-to-noise ratio (SNR), and root mean square error (RMSE) are again selected to judge the quality of the denoised EEG. The formula is as follows:

(17) $$\begin{array}{c} SNR=10*\log_{10}\left(\frac{\sum_{i=1}^{N}x^{2}\left(i\right)}{\sum_{i=1}^{N}{\tilde{x}}^{2}\left(i\right)}\right),\\ RMSE=\sqrt{\frac{1}{N}\sum_{i=1}^{N}{\left(x\left(i\right)-\tilde{x}\left(i\right)\right)}^{2}}. \end{array}$$

Among them, N, *x*(*i*), $\tilde{x}\left(i\right)$ are the length of the EEG, the original EEG, and the EEG after noise reduction. Randomly select 100 groups of EEG and obtain the signal-to-noise ratio and the root mean square error, respectively, and calculate the average value after the denoising experiment. The experimental results are shown in Table 1.

It can be seen from Table 1 that the improved soft threshold denoising method has the largest SNR, while the RMSE is the smallest, and the denoising effect is the best.

### 4.2. EM and Energy Spectrum Feature Extraction Results

**Figure 6:**
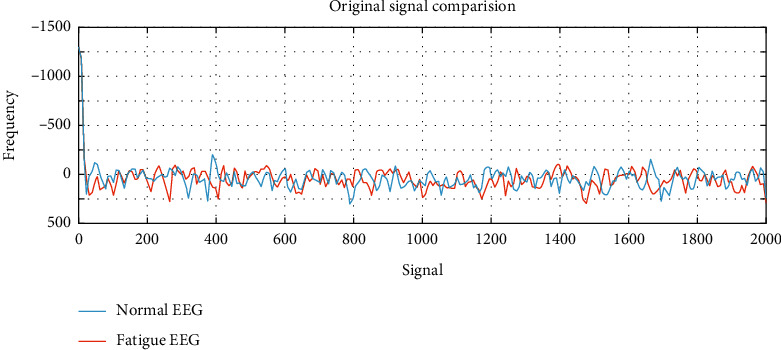
Original signal comparison.

**Figure 7:**
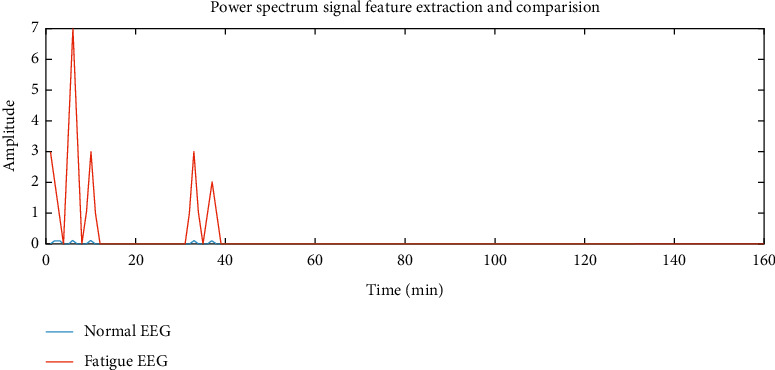
Power spectrum signal feature extraction and comparison.

**Figure 8:**
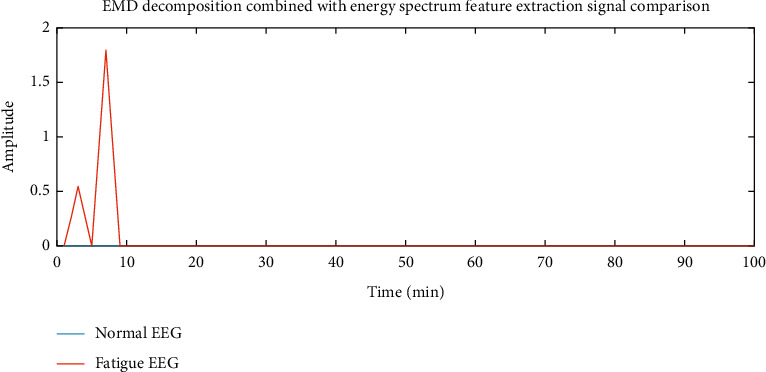
EMD decomposition combined with energy spectrum feature extraction signal comparison.

In this experiment, the EEG data of 5 subjects is selected as the training sample set, and the remaining 1 data is used for feature extraction and classification experiments as the sample test. And any set of normal signals and fatigue signals recorded in the driving fatigue experiment were selected for data analysis. In the experiment, the preprocessed signal is selected for feature extraction. After EMD decomposition and energy spectrum feature extraction, the data is compressed to 84 feature points; after power spectrum feature extraction, the EEG data is compressed to 144 feature points. The preprocessed EEG is compared with the signal after the two feature extraction algorithms, and the results are shown in Figures 6–8.

It can be seen from Figure 6 that before the feature extraction, the signals of the normal signal and the fatigue signal are entangled, which is very difficult to judge. It can be seen from Figure 7 that after using the power spectrum to perform feature extraction on the signal, it can be seen that the low-frequency range of the EEG in the fatigue state fluctuates more, and it can be easily distinguished from the normal EEG. It can be seen from Figure 8 that after EMD decomposition combined with energy spectrum for feature extraction, the energy difference feature in the low frequency range is more obvious than the power spectrum feature. Therefore, it can be assumed that the feature extraction method combined with EMD decomposition and energy spectrum is more suitable than the power spectrum. EEG recognition of driving fatigue.

Want to understand the pros and cons of the two feature extraction algorithms, and analyze the optimization effect of the PSO-H-ELM algorithm, so I select the EEG data of 5 participants as the training set, and the EEG data of the remaining participants as the test set. The experimental result is the operation of different subjects as the test set, as shown in Table 2.

It can be seen from Table 2 that although the test results of different subjects are biased, it can be found from the average accuracy rate that the features extracted using EMD decomposition combined with energy spectrum are using SVM, KNN and PSO-H-ELM three classifiers In the case, the classification effect is better than using the power spectrum feature extraction method. At the same time, the results show that the algorithm can achieve the best classification effect after using the algorithm of EMD decomposition combined with energy spectrum for feature extraction.

### 4.3. Classification Performance of Different Algorithms

Select 240 sample data of 1 participant as the test set, and 1200 sample data of the remaining 5 participants as the training set. The purpose of this arrangement is to avoid confusion between training and test data. The results are shown in Table 3.

It can be seen from Table 3 that the accuracy of 72.32%, 71.47%, 89.25%, 93.17%, 73.85%, and 70.79% were obtained by using KNN. The accuracy rates of support vector machines are 77.25%, 74.67%, 88.32%, 94.83%, 63.29%, and 73.33%, respectively. The accuracy rates of ELM are 70.85%, 66.83%, 86.25%, 88.77%, 58.25% and 67.75%, respectively. The accuracy rates of H-ELM are 78.17%, 81.08%, 89.24%, 89.38%, 71.78%, and 74.24%, respectively. Finally, the accuracy of 78.91%, 83.85%, 91.25%, 91.78%, 73.33%, and 76.75% were obtained using PSO–H-ELM. The accuracy of the algorithm changes greatly when different subjects are used as training samples. Therefore, it is difficult to directly determine from Table 3 which classification algorithm has the best effect. To make the comparison of the experimental results more intuitive, the average classification accuracy rate of 5 classifiers is calculated, as shown in Table 4.

It can be seen from Table 4 that the average accuracy of the PSO–H-ELM algorithm is the highest, indicating that the proposed PSO–H-ELM algorithm is superior to other algorithms in driving fatigue detection. By comparing the performance of H-ELM and the proposed PSO–H-ELM in different situations, it can be found that the change of training data does not affect the optimization of the H-ELM algorithm by the PSO algorithm.

To confirm the results of the experiment, paired *t*-tests are used to test the accuracy of PSO-H-ELM to see if it has obvious advantages compared to other classifiers. The results of the statistical test are shown in Table 5.

It can be seen from Table 5 that the PSO–H-ELM algorithm has only about 4% advantage compared with the average accuracy of the KNN algorithm and the SVM algorithm. The gap is not as large as expected, but it is used as a new algorithm for fatigue EEG. The detection is indeed more suitable compared to the two traditional algorithms. When comparing the PSO–H-ELM algorithm to the ELM algorithm and the H-ELM algorithm, it can be found that the advantages of the PSO–H-ELM algorithm are very obvious and stable. PSO–H-ELM is an optimized algorithm for H-ELM. The classification and recognition of EEG is much more accurate than that of H-ELM algorithm. Compared with traditional mainstream algorithms, the PSO–H-ELM algorithm is more suitable for the classification of driving fatigue EEG.

## 5. Discussion

(1) Living environment: the place of residence is too far from the work place; there are too many housework or the husband and wife are not in harmony; the mental burden is heavy; (2) Sleep quality: going to be too late, too little sleep time; poor sleep effect; noisy sleep environment cannot guarantee sleep quality. (3) In-car environment: poor air quality, poor ventilation; too high or too low temperature; bad noise and vibration; improper seat adjustment; tense relationship with fellow passengers. (4) Environment outside the car: driving in the afternoon, evening, early morning, and late at night; poor road conditions; good road conditions, single situation; driving in windy, rainy, foggy, snowy weather; poor traffic environment or crowded traffic conditions. (5) Operating conditions: long-term, long-distance driving; too fast or too slow; too limited time to reach the destination. (6) Physical condition: poor physical strength and endurance; decreased visual and hearing ability; weak physical strength or suffering from certain chronic diseases; taking drugs that are contraindicated in driving vehicles; special physiological periods for women (menstrual period, pregnancy). (7) Driving experience: low technical level and unfamiliar operation; short driving time and little experience; poor safety awareness.

Driving at high speed can easily make the driver nervous. Driving at a slightly lower speed than normal without affecting traffic can reduce mental stress and fatigue. At the same time, we try to reduce overtaking when driving, reduce emergency braking, and other actions, which can also reduce fatigue. When you feel sleepy during driving, you can also open the window to ventilate (to prevent endogenous oxygen deficiency), play different styles of music, chew gum, apply wind oil essence, and body spirit dew is more effective to prevent fatigue driving. A must-have, when you are sleepy, apply on the Renzhong acupoint, and take a deep breath to keep your brain awake and reduce fatigue. Under the premise of not affecting safety, you can stretch your fingers, shake your arms, twist your neck, and look as far away as possible. Always staring at the center line will cause visual fatigue.

If you drive a motor vehicle for more than 4 hours continuously, you should stop and rest, and the rest time should not be less than 20 minutes. Passenger vehicles, heavy-duty trucks, and semitrailer tractors used for highway operation shall install and use driving recorders that meet national standards. Traffic police can check the driving speed, continuous driving time, and other driving status information of the motor vehicle.

## 6. Conclusions

In daily life, the driver cannot always drive the vehicle in a stable state. When the driver finishes a day's work, his brain power becomes exhausted, and it is very easy to experience fatigue when driving a vehicle. To avoid the occurrence of fatigue driving, this article uses the stable and reliable characteristics of EEG, collects EEG through the brain-computer interface, and processes and analyzes it, to achieve the purpose of judging and early warning of fatigue driving. This paper implements a feature extraction method that combines empirical mode decomposition and energy spectrum: the data is decomposed into multiple IMF components through empirical mode decomposition, and then the energy spectrum is used to extract features for each IMF component, and these components are combined at the feature points to construct the feature vector of EEG. The experiment uses the new method and the power spectrum method to extract the features of EEG and uses a variety of classifiers for classification processing. Compare the classification experiment results of the two methods, and the better feature extraction effect of the new method is verified. This research still has certain limitations. For example, there are still some differences between simulated driving and actual driving, the simulated driving time is not long enough, the time when drivers of different physiques are fatigued under different road conditions, and the characteristics of fatigue will be different. There are still many problems in the development of driving fatigue research, such as real-time problems, contact problems, and cost-effectiveness of detection devices.

## Figures and Tables

**Table 1:** SNR and RMSE results after denoising with different threshold methods.

Evaluation index	Threshold method		
	Hard threshold	Soft threshold	Improve soft threshold
SNR	14.49	14.36	18.42
RMSE	276.32	278.96	197.16

**Table 2:** Different feature extraction algorithms using SVM, KNN, and PSO-H-ELM classifiers to classify the accuracy (%).

Feature extraction algorithm	Classification algorithm	1	2	3	4	5	6	Average accuracy
Power spectral density	SVM	81.43	83.59	94.38	89.76	70.79	74.13	83.33
	KNN	77.39	77.57	97.33	87.57	88.58	89.32	86.28
	PSO–H-ELM	81.43	80.76	97.73	97.06	85.75	85.26	88.83

EMD	SVM	90.75	85.38	88.25	100.00	97.29	95.67	93.62
	KNN	77.25	83.33	88.71	100.00	97.25	95.67	90.94
	PSO–H-ELM	89.60	88.85	94.25	69.57	99.85	98.23	94.24

**Table 3:** Classification accuracy using different training samples (%).

	Subject 1	Subject 2	Subject 3	Subject 4	Subject 5	Subject 6
KNN	72.32	71.47	89.25	93.17	73.85	70.79
SVM	77.25	74.67	88.32	94.83	63.29	73.33
ELM	70.85	66.83	86.25	88.77	58.25	67.75
HELM	78.17	81.08	89.24	89.38	71.78	74.24
PSO-HELM	78.91	83.85	91.25	91.78	73.33	76.75

**Table 4:** The average classification accuracy of the algorithm (%).

	KNN	SVN	ELM	HELM	PSO-HELM
Accuracy	79.31	79.31	74.08	81.67	83.12

**Table 5:** The correct rate of each classifier *t*-test test.

	Pairing bias					t	df	Sig.(2-tailed)
	Mean	Standard deviation	Mean error	95% confidence interval				
				Lowest	Highest			
P–KNN	3.820	4.985	1.949	-1.192	8.832	1.949	5	0.103
P-SVM	3.820	4.923	2.006	-1.338	8.821	1.834	5	0.119
P-ELM	9.833	4.576	1.867	4.791	14.404	5.133	5	0.004
P-HELM	1.453	0.683	0.279	0.741	2.175	5.127	5	0.001

## Data Availability

The data that support the findings of this study are available from the corresponding author upon reasonable request.
